# Operando Characterization and Theoretical Modeling of Metal|Electrolyte Interphase Growth Kinetics in Solid-State Batteries. Part I: Experiments

**DOI:** 10.1021/acs.chemmater.2c03130

**Published:** 2023-01-20

**Authors:** Edouard Quérel, Nicholas J. Williams, Ieuan D. Seymour, Stephen J. Skinner, Ainara Aguadero

**Affiliations:** † Department of Materials, Imperial College London, Exhibition Road, LondonSW7 2AZ, U.K.; ‡ Instituto de Ciencia de Materiales de Madrid, ICMM-CSIC, Sor Juana Ines de La Cruz 3, 28049Madrid, Spain

## Abstract

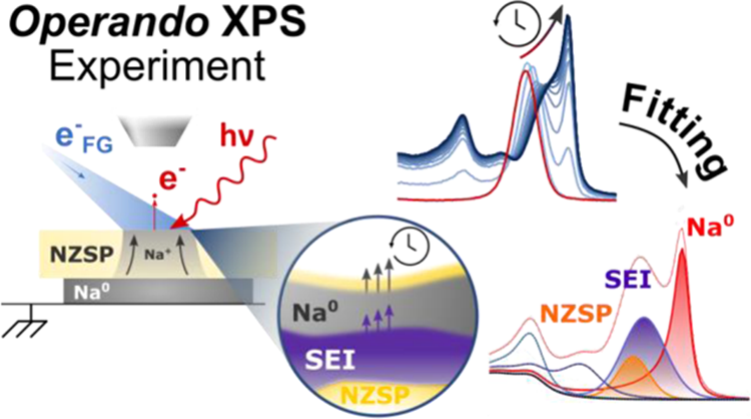

To harness all of the benefits of solid-state battery (SSB) architectures in terms of energy density, their negative electrode should be an alkali metal. However, the high chemical potential of alkali metals makes them prone to reduce most solid electrolytes (SE), resulting in a decomposition layer called an interphase at the metal|SE interface. Quantitative information about the interphase chemical composition and rate of formation is challenging to obtain because the reaction occurs at a buried interface. In this study, a thin layer of Na metal (Na^0^) is plated on the surface of an SE of the NaSICON family (Na_3.4_Zr_2_Si_2.4_P_0.6_O_12_ or NZSP) inside a commercial X-ray photoelectron spectroscopy (XPS) system while continuously analyzing the composition of the interphase operando. We identify the existence of a solid electrolyte interphase at the Na^0^|NZSP interface, and more importantly, we demonstrate for the first time that this protocol can be used to study the kinetics of interphase formation. A second important outcome of this article is that the surface chemistry of NZSP samples can be tuned to improve their stability against Na^0^. It is demonstrated by XPS and time-resolved electrochemical impedance spectroscopy (EIS) that a native Na*_x_*PO*_y_* layer present on the surface of as-sintered NZSP samples protects their surface against decomposition.

## Introduction

Among the avenues considered to improve the performance and safety of Li-ion batteries, the elimination of hazardous liquid electrolytes and their replacement by solid electrolytes (SEs) in cell architectures called solid-state batteries (SSBs) provides an attractive option.^1^ Indeed, if employing high-capacity alkali metal negative electrodes, SSBs offer a solution to simultaneously increase the energy density, power density, and safety of cells. While it was initially believed that SSBs would also benefit from intrinsic long-term stability,^2^ it has later been demonstrated that the lifetime of SSBs is highly impacted by degradation at the electrode|SE interface.^3−5^ Some of these issues are related to the electrochemical stability of the SE with regard to electrode materials and the formation of interphases upon decomposition of the SE. This initial instability is not necessarily an issue if a stable solid electrolyte interphase (SEI) can be formed, such as at the interface between graphite and optimized liquid electrolytes in conventional Li-ion cells.^6^ The decomposition of the SE against an alkali metal leads to the formation of interphases whose electronic properties will dictate its growth:^7^ (a) if a majority of the decomposition products are electronically insulating, the growth of the SEI will eventually stop, and if it does not form a large barrier to ionic migration, its impact on the power performance of a cell may be tolerable, or (b) if the decomposition products are electronically conducting, the growth of the mixed ionic electronic conducting (MIEC) interphase will be uninterrupted until all of the SE is consumed and a short-circuit occurs. This latter interphase type is not compatible for SSB with long-lasting performance. Having access to the chemical composition of the interphase is essential in determining which type of interphase is produced and whether stability will be reached in a cell.

X-ray photoelectron spectroscopy (XPS) is an excellent surface characterization technique for chemical composition analysis. Analyzing the composition of a buried interface is however a challenge because of the limited depth resolution of XPS. The limited depth resolution of XPS is due to the nature of the measurement which relies on the collection of photoelectrons which escape from a sample surface after traveling a short distance away from the atomic nucleus they initially bounded with (typically within a depth of less than 10 nm for photoelectrons excited by an Al Kα source and traveling through Na metal). Recently, a variety of *in situ*(3,8,9) and operando techniques^10,11^ have been developed to address this problem. For all of them, the idea is to make the alkali metal layer on the surface of the SE thin enough to let photoelectrons emitted by the SE (and possibly by interphases) go through the metal overlayer. To produce the alkali metal layer, one technique consists in plating it on the surface of the SE from a counter electrode composed of the same alkali metal while analyzing the interphase products operando.^10^ What enables the plating in that case is the provision of low-energy electrons to the surface of the SE from the electron flood gun present in any XPS instrument. This operando XPS technique has the additional benefit of replicating the conditions which occur during the first charge of cells with zero excess capacity on the negative electrode (also sometimes referred to as “anode-free” cells).^12^ While this technique has already proven its efficacy at characterizing the composition of interphases, the extent of information that can be extracted from it (such as the growth rate behavior of the alkali metal layer) has not yet been fully appreciated. The objective of this study is to present the depth of information that can be extracted from this operando protocol. The results are separated into two paired articles (Part I: Experiment; Part II: Theory).^13^

In Part 1, the electrochemical stability of Na metal (Na^0^) on the surface of a sodium conducting SE of the NaSICON family (Na_3.4_Zr_2_Si_2.4_P_0.6_O_12_, further referred to as NZSP) is studied. NZSP is chosen for this study because of its high ionic conductivity that makes it a promising candidate SE,^14^ but its stability against Na^0^ is still debated. Theoretical density functional theory (DFT) calculations predict that Na_3_Zr_2_Si_2_PO_12_ (the closest phase on the convex hull of the NaSICON compositional space defined by Na_1+*x*_Zr_2_Si*_x_*P_3–*x*_O_12_, 0 ≤ *x* ≤ 3) is unstable at 0 V against Na/Na^+^ and should form an interphase composed of Na_2_ZrO_3_, Na_4_SiO_4_, Na_3_P, and ZrSi.^15−17^ The formation of a stable SEI at the Na^0^|Na_3_Zr_2_Si_2_PO_12_ was also suggested experimentally by electrochemical impedance spectroscopy and *ex situ* XPS studies.^17,18^

This study will distinguish two types of Na^0^|NZSP interfaces: the first is the interface between Na^0^ and a polished NZSP (NZPS_polished_) pellet; the second is the interface between Na^0^ and an as-sintered NZSP (NZSP_AS_) pellet. This comparison is intended to clarify the impact of the NZSP surface chemistry on its stability against Na^0^. Indeed, we demonstrated in a previous study using XPS, low-energy ion scattering (LEIS), and density functional theory (DFT) that a thin Na*_x_*PO*_y_* layer is present on the surface of as-sintered NZSP samples.^14^ This Na*_x_*PO*_y_* layer can be removed by polishing, exposing a pristine NZSP surface free from any SiC residues. The presence of this Na*_x_*PO*_y_* layer could have an impact on the interface stability because some sodium phosphate phases (*e.g.*, Na_3_PO_4_) are predicted to be stable against Na^0^ by DFT calculations.^19^ Thus, the aim of comparison between the two interfaces is to evaluate the efficiency of Na*_x_*PO*_y_* as a self-formed buffer layer. These interfaces are further referred to as Na^0^|Na*_x_*PO*_y_*|NZSP and Na^0^|NZSP_polished_.

The discussion of this first experimental part focuses on extracting information from the XPS fitting models to inform on the kinetics of interphase formation at both the Na^0^|NZSP_polished_ and Na^0^|Na*_x_*PO*_y_*|NZSP interfaces. Time-resolved electrochemical impedance spectroscopy (EIS) is also employed to evaluate the ionic resistivity of the interphases. Overall, this work introduces a framework for understanding the growth of interphases in solid-state batteries that can be implemented by researchers using widely available XPS instrumentation to study a diverse range of Na and Li solid-state battery systems.

## Experimental Section

### NZSP Synthesis

Na_3.4_Zr_2_Si_2.4_P_0.6_O_12_ powders were synthesized following a solution-assisted solid-state synthesis described in a previous publication.^14^ Pellet samples were produced from this mother powder and sintered in Pt crucibles (1285 °C, 6 h, 180 °C h^–1^ heating and cooling rates).

As-sintered pellets which have a native Na*_x_*PO*_y_* surface layer (and are hereinafter referred to as Na*_x_*PO*_y_*|NZSP) were quickly transferred to an Ar-filled glovebox for storage without further treatment to their surface. The pellets which are referred to as “polished” (NZSP_polished_) were polished with 500 grit SiC paper using ethanol as lubricating solvent, sonicated in ethanol for one minute, and then transferred to a glovebox for storage.

### XPS Data Acquisition

The Na^0^|NZSP half-cells used for the operando plating experiment were assembled inside an Ar-filled glovebox (O_2_ and H_2_O levels below 1 ppm). Circular Na^0^ counter electrodes were punched from a freshly prepared Na^0^ foil for each new half-cell (Na metal rod, 99%, Alfa Aesar). The surface of the Na^0^ electrode was mechanically cleaned using the blade of a scalpel before being pressed against one side of the NZSP pellet. These Na^0^|NZSP half-cell were transferred to the XPS instrument (Thermo Fisher Scientific Kα XPS system) using a vacuum transfer vessel (Thermo Fisher Scientific XPS Vacuum Transfer Module).

XPS spectra were collected at room temperature with a monochromated Al Kα source (1486.6 eV) operating at a power of 72 W (6 mA × 12 kV). The analysis area is an ellipsoid of dimensions *ca.* 400 μm × 800 μm. As described in the Introduction Section, the charge-compensating flood gun (FG) of the instrument was diverted from its intended use and employed to supply electrons for the Na^0^ plating reaction on the NZSP surface. A specificity of this instrument is that charge compensation relies on a dual-mode flood source (electrons and Ar^+^ ions) which are not independently controlled. A recent publication demonstrated that the bombardment of the interface by Ar^+^ ions during plating can impact the interphase composition.^19^ To minimize the Ar^+^ ion flux reaching the interface, the extractor voltage of the instrument was reduced to 30 V following the recommendations of a previous study.^20^ The Na^0^ plating rate was controlled by optimizing the FG parameters: the beam voltage was set to 3 V, and the current was set to 30 μA (the actual electronic current reaching the sample surface was measured using a Faraday cup to be ∼4.8 μA; the Ar^+^ current reaching the surface is ∼10 nA). The base pressure of the instrument (FG off) is typically around 1 × 10^–9^ mbar and rises to around 1 × 10^–8^ mbar when the FG is activated. The change in pressure is related to the introduction of a small volume of Ar gas in the analysis chamber associated with the design of the dual-mode flood source.

Core-level spectra were measured using a pass energy of 20 eV, at a resolution of 0.1 eV, and an integration time of 50 ms/point. A compromise between the speed of acquisition and the quality of the data had to be determined: short iterations are required because the continuous plating of Na^0^ leads to a rapid attenuation of the interface signals, but iterations should be long enough to detect chemical shifts in low-intensity signals. The adequate FG current was found empirically by testing different currents: the charge-compensating electrons either serve to compensate holes generated by the photoemission process or can participate in the Na metal plating reaction. This explains why the experiment was monitored for 4 h although the flood gun current is relatively large. Additional details about the flood gun settings and the Na metal plating rate can be found at the end of the Supporting Information.

### XPS Data Fitting

All spectra were fitted using the algorithms implemented in the CasaXPS software. Shirley backgrounds were employed for all core-level signals with minimal inelastic backgrounds (*i.e.*, Zr 3d, Si 2p, P 2p). For Na 1s regions, plasmon resonances produce a significant inelastic background and a three-parameter Tougaard background function was employed (U 4 Tougaard: B,33,0.8,0) where the intensity of the parameter B was adapted for each fit.

Most peaks were fitted using a symmetric LA (1.53,243) line shape (a Voigt function). This line shape cannot be used to model the Na metal peak, which has a tail on the high-binding-energy (BE) side of the peak. An exact procedure to model asymmetric peaks in XPS is still debated.^21^ The only asymmetric peak shape with a theoretical basis is the Doniach Sunjic (DS) one. However, a problem with the DS function is that its asymptotic form means it integrates to infinity. The extracted peak area is therefore dependent on the defined energy region. In a recent review, Major et al. propose a series of solutions to address the asymmetry problem, including the use of finite Lorentzian (LF) functions to overcome the problem of nonintegrability of the DS function.^21^ For the main Na metal peak, an LF (0.58,1.17,200,80) line shape was employed.

In some figures (*e.g.*, Figure 5), the fraction of the total Zr 3d signal emitted by the interphase species is introduced and is calculated as
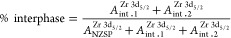
where ,  and  correspond to the area of the Zr 3d_5/2_ peaks of the interphase 1, interphase 2, and NZSP phases in the peak fitting models used in Figures 3 and 4.

### Cell Assembly and Electrochemical Impedance Spectroscopy (EIS)

Na metal films were freshly prepared for each assembled cell in an Ar-filled glovebox. A clean piece of Na^0^ was cut (Na cubes, 99.9%, Sigma-Aldrich), then pressed flat in a low-density polyethylene (LDPE) plastic bag to a thickness of ∼150 μm. Circular electrodes were then punched from this foil. Their surface was mechanically cleaned using the blade of a scalpel. The Na metal electrodes were then placed on both sides of an NZSP pellet and the Na|NZSP|Na stack was pressed with a uniaxial pressure of around 10 MPa. The symmetrical cells were then placed in 2032-type coin cells. Battery-grade Al foil was used as a nonalloying current collector between the Na^0^ electrode and stainless-steel casing.

Impedance spectra were measured on a Biologic SP-240 potentiostat with an excitation amplitude of *V*_A.C._ = 50 mV in the frequency range of 7 MHz to 5Hz, with a 20-cycle integration period at each frequency and a one cycle delay after each frequency jump. Results were fitted using RelaxIS3 (Rhd Instruments).

## Results and Discussion

### Operando Plating of Na^0^ on NZSP Surfaces

**Figure 1:**
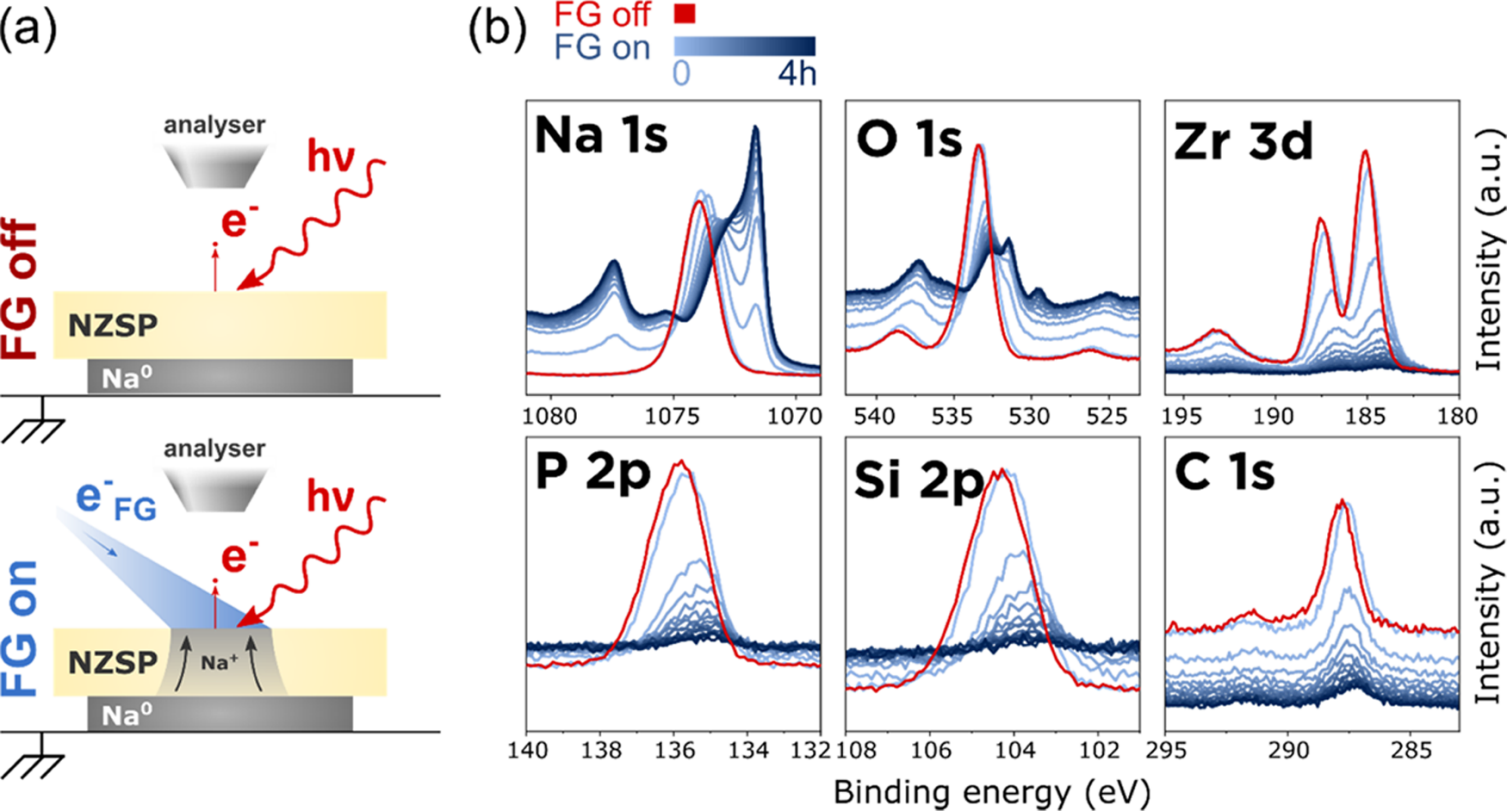
Demonstration of the working principle of the operando plating of Na^0^ inside the XPS. (a) Schematic representation of the two XPS analysis configurations: when the FG is off, the chemical composition of the bare NZSP surface is analyzed; when the FG is on, Na^0^ can plate on the top surface of NZSP and the changes in chemical composition are monitored operando; (b) evolution of selected core-level regions with increasing Na^0^ plating time. An initial set of data (in red) was measured with the FG off (reference signal from NZSP_polished_). The following spectra (in shades of blue) were measured in an iterative loop (each iteration lasted 18 min and 11 s).

The working principle of the operando plating of Na^0^ on the NZSP using the flood gun of an XPS instrument is schematically represented in Figure 1a. The results presented in this section were obtained with a polished NZSP sample (NZSP_polished_).

The analysis sequence starts with the FG initially turned off. A first set of survey and core-level spectra are measured and constitute a reference for the bare NZSP surface prior to any plating or interphase formation (shown in red in Figure 1). The FG is then turned on and core-level spectra are measured over 4 h in an iterative loop with each acquisition cycle (*i.e.*, one set of Na 1s, Zr 3d, P 2p, Si 2p, O 1s, and C 1s core-level data) lasting 18 min and 11 s. The successive sets of XPS spectra are presented in Figure 1 in shades of blue, from light blue for the first cycle (0 h of plating) to dark blue for the last cycle (around 4 h of plating).

A qualitative description of Figure 1 reveals that: (1) Na^0^ was successfully plated on the surface of NZSP using the FG; this is indicated by the growth of an intense XPS peak at 1071.8 eV (whose attribution to Na^0^ will be detailed later); (2) several new peaks appear in the Na 1s and O 1s core-level regions as plating progresses; (3) the intensity of the Zr 3d, P 2p, Si 2p, and C 1s signals decreases as plating progresses, which confirms that an overlayer is growing on top of NZSP; (4) a change in the shape of the Zr 3d signal with a tail to lower binding energies in comparison to the reference sample is observed as plating progresses; (5) the rate at which the Na metal peak grows is rapid in the first cycles and slows down subsequently; (6) all core-level spectra experience a shift to lower binding energy between the flood gun off and flood gun on condition; and (7) a continuous shift to lower binding energies (BE) of the NZSP peaks (in the Na 1s region and the Na Auger peak in the O 1s region) is observed.

To construct a physically meaningful surface model to fit the XPS data from Figure 1, the XPS signature of Na metal needs to be isolated first. For this purpose, an XPS analysis of a pristine Na metal foil was conducted.

### XPS Signature of Pristine Na^0^

The XPS signature of Na metal was obtained by analyzing separately the surface of a Na metal sample (99%, Alfa Aesar). The sample was prepared inside an Ar-filled glovebox and transferred to the XPS instrument via a vacuum transfer vessel. Despite having taken such precautions, a passivation layer was found on the surface of the sample (see Figure S1). To remove the passivation layer, the Na metal foil was sputter-cleaned using a 2 keV Ar^+^ ion gun inside the XPS chamber for a total sputtering time of 3 h. The removal of surface contaminants was confirmed in the survey spectrum by the disappearance of the signals in the O-KLL and C 1s regions and by the metallic Fermi edge in the valence band region.

**Figure 2:**
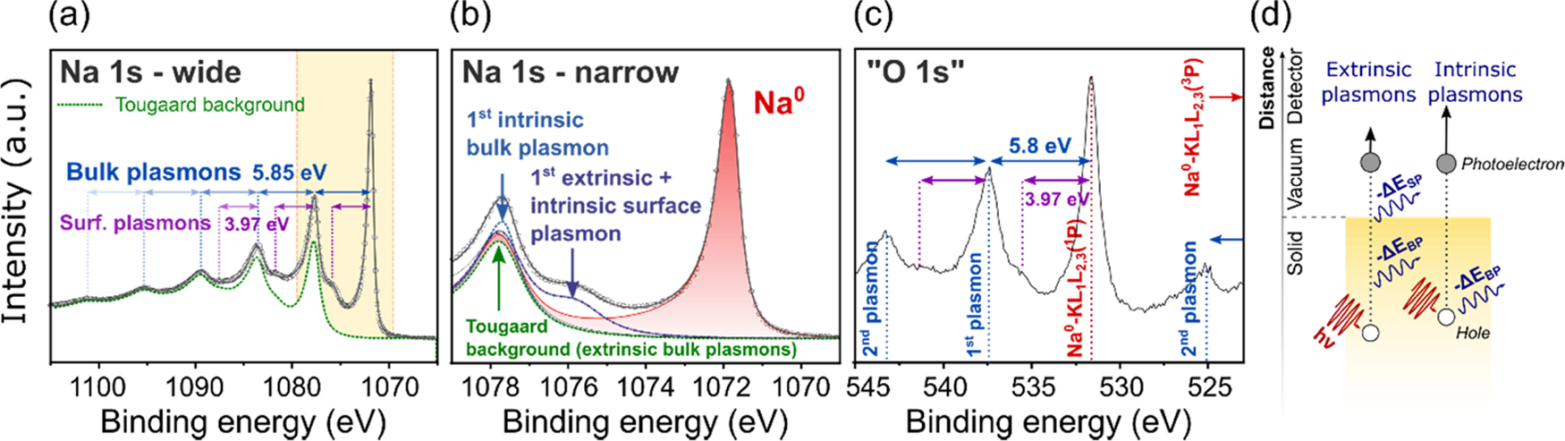
Fitting model of sputter-cleaned Na metal. (a) Na 1s region measured over a wide BE range. The yellow box indicates the region which is magnified in (b); (b) fitting model of the Na 1s region. Several peaks were added to model the background. (c) Identification of the peaks observed in the “O 1s” region. (d) Schematic showing different origins of plasmon excitations.

Figure 2 shows XPS signals of the Na 1s and “O 1s” regions of the sputter-cleaned Na metal sample. The Na 1s region was scanned over a wide BE range, Figure 2a, to collect multiple plasmon peaks. Figure 2b provides a narrower range view of the Na 1s region centered around the main Na^0^ peak. Figure 2c shows the XPS signals in the region where O 1s signals are typically found (between 520 and 545 eV). The region is called the “O 1s” region in relation to Figure 1, but it should be noted that the peaks are primarily caused by Na metal Auger photoelectrons and their plasmon losses.

Figure 2a shows that the main Na^0^ peak (at 1071.83 eV) is followed by a series of periodic plasmon peaks. A plasmon is a collective oscillation of the valence electrons of a metallic sample.^22^ The peaks which appear periodically at higher BE from the main Na^0^ peak are photoelectrons that have lost one or several quanta of energy to excite plasmon resonances. Photoemission can excite bulk and surface plasmons in metallic samples. In Figure 2a, bulk plasmons produce strong peaks at integral multiples of Δ*E*_BP_ = 5.85 eV from the main Na^0^ peak. The first surface plasmon is observed at Δ*E*_SP_ = 3.97 eV from the main Na^0^ peak (the experimental ratio Δ*E*_BP_/Δ*E*_SP_ = 1.47 is close to the theoretical value of √2). Other surface plasmons are observed at multiples of *n*. Δ*E*_BP_ + Δ*E*_SP_ (with *n*, an integer number) which corresponds to the case when a photoelectron excites *n* bulk plasmons and a surface plasmon as it exits to the surface of the sample.

Barrie and Street analyzed the XPS signals of sodium metal and sodium oxide in 1975, but a fitting model for the Na 1s region of Na^0^ and its inelastic background are, to the best of our knowledge, not available in the literature.^23^ Because plasmon resonances introduce a strong modification of the inelastic background up to 50 eV below the main peak, appropriate peak fitting requires the use of a Tougaard background instead of the more common Shirley background.^24^ The Tougaard background function was parameterized from the wide-region XPS scan (Figure 2a): the function replicates the periodicity of the bulk plasmon losses, and the background reaches the baseline at 30 eV higher BE from the main peak. Figure 2a shows that the Tougaard function models the intensity of the background well away from the main peak (at 15 eV higher BE upwards), but that it underestimates the intensity of the first and second plasmon peaks. This is because the Tougaard function employed does not model intrinsic plasmons but only extrinsic ones.^25,26^ Intrinsic plasmons are excited at the photoemission site and simultaneously to the photoemission event whereas extrinsic plasmons are excited away from the photoemission site as photoelectrons travel through the metal. Figure 2d schematically represents the difference between intrinsic and extrinsic plasmons. In this work, the contribution from intrinsic plasmons was modeled using separate peaks indicated in Figure 2b. It is important to note that these peaks are part of the inelastic background of the Na^0^ peak and are not primary photoelectrons. Figure 2b also shows that the main asymmetric Na^0^ peak was modeled using a finite Lorentzian (LF) function. An LF function was preferred over a Doniach Sunjic function to limit the tail of the metal peak and enable the integration of its area without introducing a cutoff.^21^ The fitting model parameters are presented in Table S1.

Figure 2c provides an identification of the peaks in the “O 1s” region. The most intense peak at 531.6 eV is a Na–KL_1_L_2,3_(^1^P) Auger peak. It is important to notice that the position of this Auger peak differs between metallic sodium and sodium oxides.^23^ For instance, the Na–KL_1_L_2,3_(^1^P) Auger from NZSP is located at 7.3 eV higher binding energies (around 538.9 eV in Figure 1). Auger photoelectrons can also excite plasmons, and the plasmon peaks are observed with the same periodicity as in the Na 1s region (at multiples of Δ*E*_BP_ = 5.8 eV and Δ*E*_SP_ = 3.97 eV). The peak observed at 525 eV is the second plasmon peak of Na–KL_1_L_2,3_(^3^P) Auger photoelectrons.

To assess the reactivity of Na metal with residual gases inside the XPS chamber, the Na metal foil was left for 6 h under ultrahigh vacuum (5 × 10^–9^ mbar) with all guns off (X-ray, sputter, charge compensation). The XPS survey and core-level signals of the Na metal sample after the 6 h pause are presented in Figures S1 and S2. The Na 1s and O 1s signals clearly indicate the formation of a passivation layer on the Na^0^ surface. Thus, even under ultrahigh-vacuum conditions, a passivation layer can form on the surface of Na metal films in a few hours. It is important to note that the operando metal plating experiment (Figure 1) typically lasts around 4 h, which leaves enough time for a passivation layer to form on the surface of the plated Na metal.

### Fitting Model for the Na^0^|NZSP_polished_ Interface

**Figure 3:**
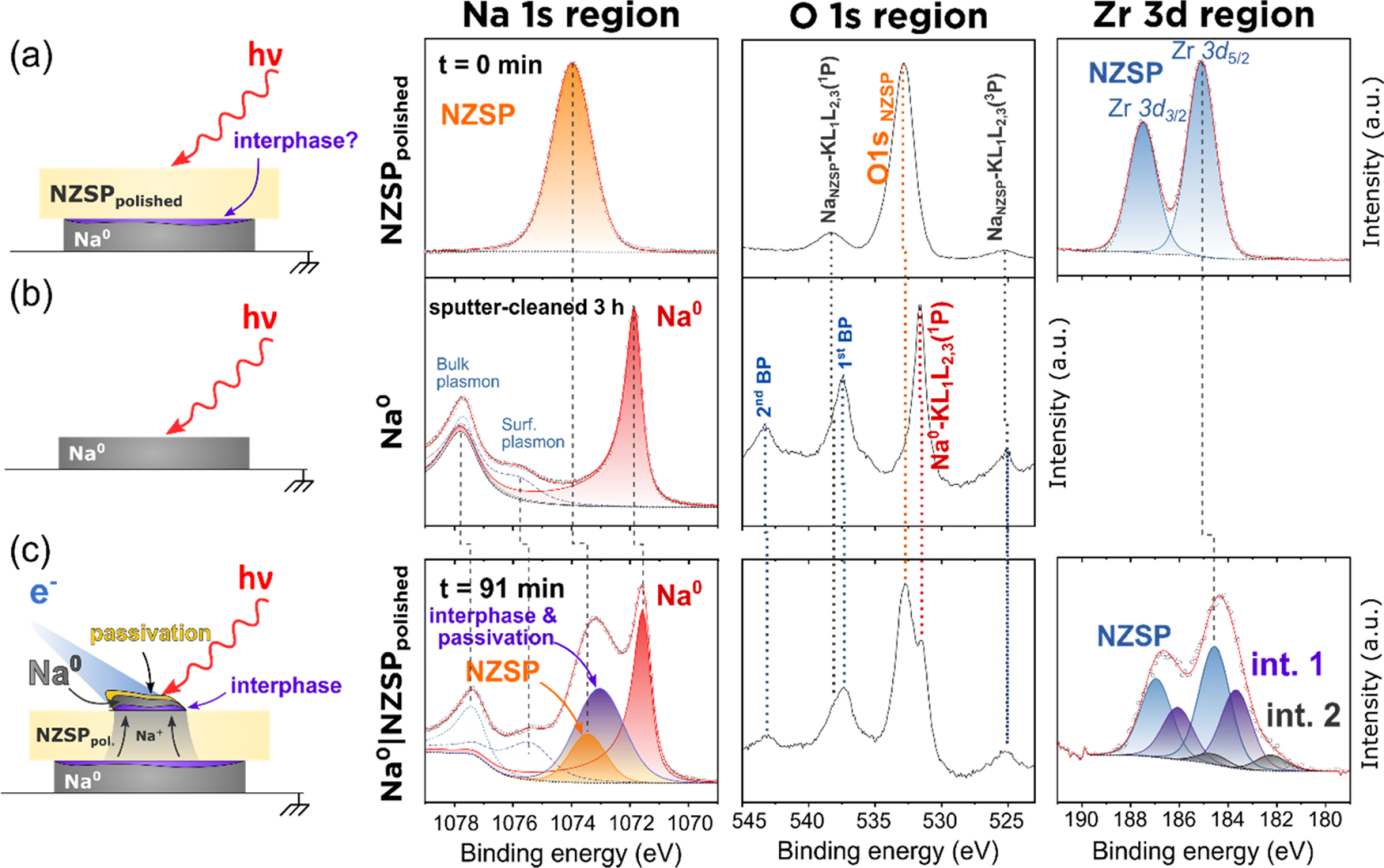
Fitting model for the Na^0^|NZSP_polished_ interface. (a) XPS fitting model of a polished NZSP surface before the initiation of plating (FG off); (b) XPS fitting model of a sputter cleaner Na metal foil; (c) XPS data of a selected iteration (*t* = 91 min of plating) and the corresponding fitting model for the Na^0^|NZSP_polished_ interface. Fitting parameters and constraints are listed in Tables S2 and S3. The schematics on the left are graphical aids, and the various layers are not at scale.

The Na metal fitting model previously established is used in this section as a reference to identify the new peaks which appeared during the operando plating of Na^0^ on top of NZSP_polished_ (experiment described in Figure 1). As the most significant changes affected the Na 1s, O 1s, and Zr 3d signals, these core levels are discussed in detail in Figure 3. Figure 3 compares the XPS signals of three samples: the Na^0^|NZSP_polished_ interface after 91 min of plating, the reference sputter-cleaned Na^0^ surface, and a reference NZSP_polished_ surface.

### Na 1s

The Na 1s region of Na^0^|NZSP_polished_ is constituted of five peaks. Three of these peaks correspond to the Na 1s signature of Na^0^ (see Figure 2) and can therefore be assigned to the freshly plated Na^0^ layer: the most intense peak is the asymmetric Na^0^ peak at 1071.9 eV (Figure 3, red); it is followed by the first surface and bulk plasmon peaks, respectively, at 3.96 and 5.86 eV higher BE from the Na^0^ peak (in dashed lines). As a reminder, these plasmon peaks are part of the Na^0^ peak background and are not produced by a separate phase. In addition to these three Na^0^ signals, there is one peak corresponding to the NZSP phase (in orange) at 1073.47 eV. The area of the NZSP peak was constrained to respect the initial stoichiometry (Na/Si ratio) measured from the reference sample (NZSP_polished_). The remaining Na 1s intensity was fitted by a fifth peak (in purple). This peak is attributed to photoelectrons emitted by the interphase and/or a surface passivation layer. As discussed in the previous section, a passivation layer can form on the surface of reactive Na metal in a few hours even in an ultrahigh-vacuum environment. Future studies will have to design solutions to separate the contribution from the passivation layer and the interphase species in the Na 1s region. The full list of fitting constraints and calculated parameters can be found in Tables S2 and S3.

### O 1s

The O 1s region of the Na^0^|NZSP_polished_ interface was only qualitatively compared with that of NZSP_polished_ and Na^0^ in Figure 3 because of the large number of overlapping peaks in this region. The peaks observed in the 523–545 eV range of the Na^0^|NZSP_polished_ interface are produced by: (i) Na–KLL Auger electrons from the plated Na^0^ layer, from the NZSP phase, and from the newly formed interphase and passivation layer; (ii) plasmon peaks from the Na–KLL Auger photoelectrons; and (iii) O 1s photoelectrons from the NZSP phase and from the interphase and passivation layer. For the Na^0^ reference sample, the peak at around 525 eV corresponds to a plasmon peak of Na^0^–KL_1_L_2,3_(^3^P) Auger photoelectrons (whose main peak is at lower BE and is not observable here).

### Zr 3d

The fitting model for the Zr 3d region of the Na^0^|NZSP_polished_ interface consists of three doublets. The first of these doublets (in blue) corresponds to Zr 3d photoelectrons emitted by the NZSP phase. A tail appears in the 181–184 eV region as soon as Na^0^ plating starts, which suggests that an interphase forms. The additional doublets required to fit the tail were constrained to have the same FWHM as that of the NZSP doublet. It was found that two doublets were required to obtain a fitting model with low residuals. The chemical composition of the phases giving rise to these signals could not be unambiguously determined with this experiment. Thus, the new doublets are referred to as “int.1” and “int.2” (for “interphase 1/2”) in Figure 3. Future work will be required to confirm if one of these doublets can be assigned to Na_2_ZrO_3_ which is one of the decomposition products predicted to form at the Na^0^|Na_3_Zr_2_Si_2_PO_12_ interface from first-principles calculations.^15−17^ In addition to Na_2_ZrO_3_, ZrSi and ZrP are two other phases predicted to form at the Na^0^|Na_3_Zr_2_Si_2_PO_12_ interface by DFT. It is however unlikely that the interphase doublets correspond to these phases because their formation would result in a clear signal in the Si 2p and P 2p regions, which was not observed (see below).

### Si 2p and P 2p

The formation of an interphase at the Na^0^|NZSP_polished_ interface did not result in significant changes in the Si 2p and P 2p signals (see Figure S3). As previously mentioned, the formation of Na_3_P, ZrSi, and ZrP could not be detected although these phases are predicted to form by DFT. Because P is in a (−III) oxidation state in Na_3_P and ZrP and Si is in a (−IV) oxidation state in ZrSi, the presence of any of these phases should result in a peak at lower BE in comparison to the NZSP peaks (where P is in a (+V) state and Si is in a (+IV) state).

### Fitting Model for the Na^0^|Na*_x_*PO*_y_*|NZSP Interface and Role of Na*_x_*PO*_y_* as a Protecting Layer

The results from the previous section established that polished NZSP pellets form an interphase in contact with Na metal. This section investigates the stability of as-sintered NZSP pellets against Na^0^. As a reminder, it was established in a previous study that the surface of as-sintered NZSP samples is terminated by a thin Na*_x_*PO*_y_* layer (the sample is therefore here referred to as Na*_x_*PO*_y_*|NZSP).^14^ The aim of this section is therefore to clarify whether this layer has an impact on the stability of NZSP against Na^0^.

**Figure 4:**
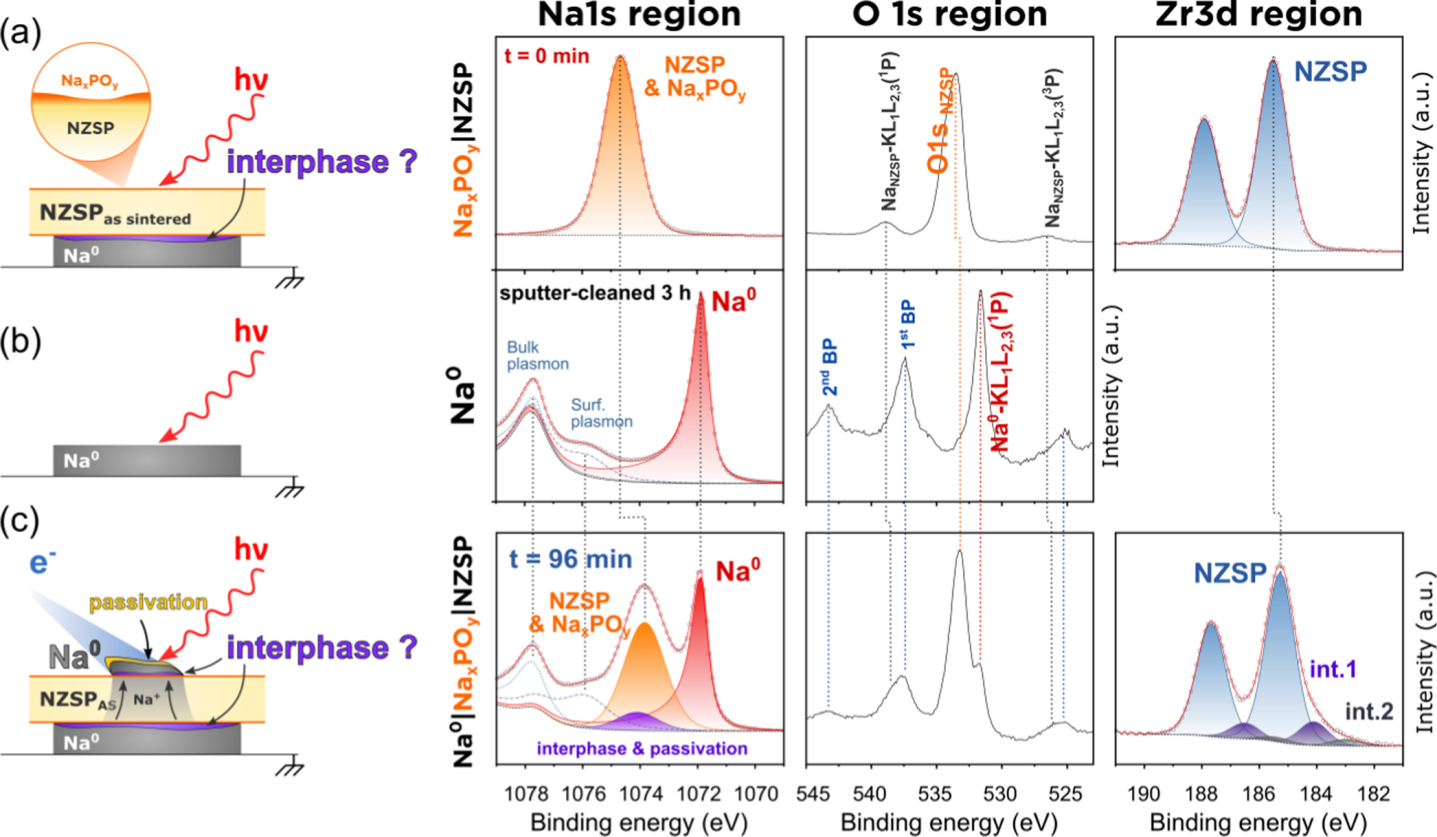
Fitting models for the Na^0^|Na*_x_*PO*_y_*|NZSP interface. (a) XPS fitting model of an as-sintered NZSP surface covered by a self-formed Na*_x_*PO*_y_* layer before the initiation of plating (FG off); (b) XPS fitting model of a sputter cleaner Na metal foil; (c) XPS data of a selected iteration (*t* = 96 min of plating) and the corresponding fitting model for the Na^0^|Na*_x_*PO*_y_*|NZSP interface. Fitting parameters and constraints are listed in Tables S4 and S5. The schematics on the left are graphical aids, and the various layers are not at scale.

The same procedure was employed to plate Na^0^ on the surface of the Na*_x_*PO*_y_*|NZSP pellet (see Figure S4). Figure 4 includes the fitting model for the Na 1s and Zr 3d core levels of a Na^0^|Na*_x_*PO*_y_*|NZSP interface after 96 min of Na^0^ plating (also included in the figure are the XPS signature of reference Na*_x_*PO*_y_*|NZSP and Na^0^ surfaces). Figure 4 shows that Na*_x_*PO*_y_*|NZSP surfaces are less reactive against Na^0^ than NZSP_polished_ surfaces. This can be observed in the Na 1s region where the intensity of the peak corresponding to interphase and surface passivation species (in purple) is much smaller than in Figure 3. This is also the case for the interphase doublets in the Zr 3d region.

No significant changes in the shape of the Si 2p and P 2p signals were observed for Na^0^|Na*_x_*PO*_y_*|NZSP in comparison to the reference signals (Figure S5). The relative shift in peak positions is discussed in the following sections and in Part II of this study.

### Evolution of the Peak Areas as a Function of Plating Time

**Figure 5:**
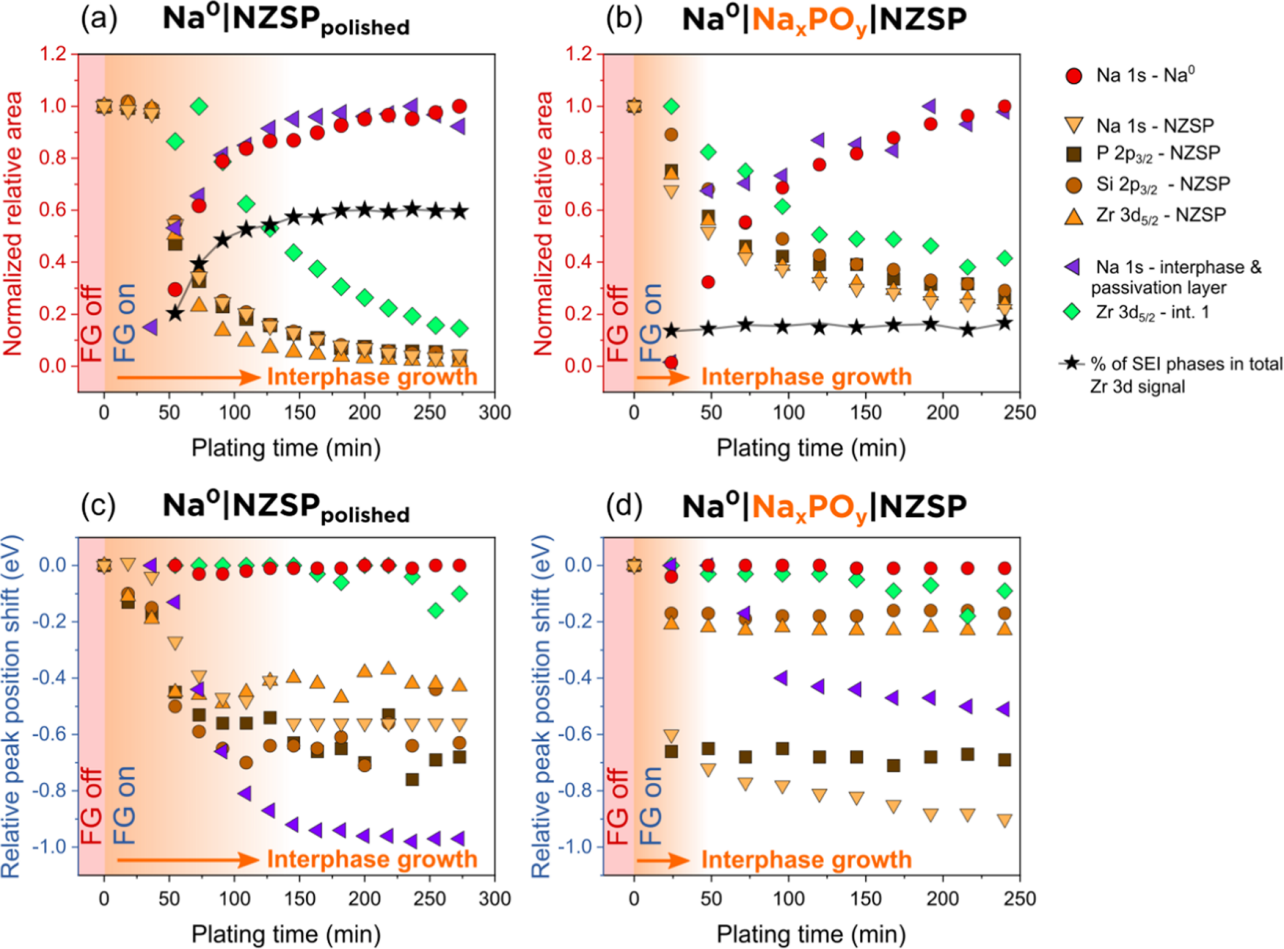
Evolution of the fitting model peak areas and peak positions as a function of plating time. Fitted peak areas (normalized to their relative maximum) as a function of plating time for (a) the Na^0^|NZSP_polished_, and (b) the Na^0^|Na*_x_*PO*_y_*|NZSP interface. Relative shifts in peak positions as a function of plating time for the (c) Na^0^|NZSP_polished_ interface and (d) the Na^0^|Na*_x_*PO*_y_*|NZSP interface. FG off/on: flood gun off/on. The orange-shaded area shows the duration of interphase growth in both cases.

Figure 5a,b plots the relative evolution of peak areas as Na^0^ plating progresses. The peaks were individually normalized relative to their maximum area over the course of the experiment. The moment when this maximum is reached differs depending on the peak: for instance, the Na^0^ peak reaches its maximum at the end of the experiment (*i.e.*, when the plated Na^0^ layer is the thickest), whereas the peaks corresponding to NZSP are most intense at the beginning of the experiment (before NZSP gets covered by an overlayer). For both the Na^0^|Na*_x_*PO*_y_*|NZSP and Na^0^|NZSP_polished_ interfaces, the growth of the Na^0^ peak can be separated into two regions: a fast initial growth (in the first 100 min of plating) followed by a slowdown in the rate of plating.

The formation of an interphase at both the Na^0^|Na*_x_*PO*_y_*|NZSP and Na^0^|NZSP_polished_ interfaces was detected by the appearance of new species in the Zr 3d region in Figures 3 and 4. The evolution of the normalized relative area of one of these peaks (“int. 1”) is included in Figure 5a,b (in purple). Figure 5a shows that the normalized area of the “int. 1” peak attenuates as plating progresses, which indicates that Na^0^ grows on top of the “int. 1” phase. The normalized relative area of the “int. 1” peak is larger than the NZSP peaks (*e.g.*, Zr 3d), which also suggests that the new phase lies on top of the NZSP phase. In contrast, for the Na^0^|NZSP_polished_ interface, the intensity of the “int. 1” peak initially increases in the first minutes of Na^0^ plating before attenuating. The rate at which the “int. 1” peak attenuates is also slower than that of the NZSP peaks. This suggests that the decomposition of NZSP and the formation of an interphase occur over a longer time on NZSP_polished_ surfaces in comparison to Na*_x_*PO*_y_*|NZSP ones.

The black stars in Figure 5a,b represent the ratio of the interphase peaks to the total signal detected in the Zr 3d region (see the Experimental Section for more details on the calculation). This ratio provides information about the progression of the decomposition reaction occurring at the interface. The decomposition reaction takes around 2 h to stabilize for the Na^0^|NZSP_polished_ interface, whereas it is almost immediate for the Na^0^|Na*_x_*PO*_y_*|NZSP interface. The fraction of the Zr 3d signal emitted by the interphase is around 15% for the Na^0^|Na*_x_*PO*_y_*|NZSP interface and up to 60% for the Na^0^|NZSP_polished_ interface. NZSP_polished_ surfaces therefore decompose to a much greater extent than Na*_x_*PO*_y_*|NZSP surfaces in contact with Na^0^.

The main conclusion of this subsection is that the NZSP surface chemistry influences its stability versus Na^0^. Na*_x_*PO*_y_* acts as a protecting layer preventing the formation of a thick interphase. Another important conclusion is that for both Na^0^|Na*_x_*PO*_y_*|NZSP and Na^0^|NZSP_polished_, the decomposition reaction eventually stabilizes, which suggests that the reaction products are electronically insulating and form a self-limiting SEI-type interphase.^4^ The formation of a self-limiting interphase is crucial to ensure the long-term stability of Na^0^|NZSP-based batteries.

### Relative Shifts of the Peak Positions as a Function of Plating Time

Figure 5c,d shows the relative shifts in the positions of peaks as plating progresses for the Na^0^|NZSP_polished_ and Na^0^|Na*_x_*PO*_y_*|NZSP interfaces. For NZSP photoelectrons, the shifts are calculated with respect to the peak position measured in the reference (FG off) experiment. The peak position is affected by the surface potential that the photoelectrons experience as they escape the surface into vacuum. In other words, if a surface potential accelerates photoelectrons when they leave the surface, the resulting peak will appear in the spectrum at a lower BE than it would normally appear without the surface potential. The surface potential is continuously changing in the operando experiment from the combined effect of Na^0^ plating and the formation of interphase. Thus, it is not surprising to observe a shift in the position of peaks in Figure 5. For the Na^0^|Na*_x_*PO*_y_*|NZSP interface (Figure 5d), an initial shift of all of the peaks happens in the first few minutes of plating, but the peaks then stop shifting for the rest of the experiment. The relative shifts of the Na 1s and P 2p peaks are more pronounced (−0.6 to −0.8 eV) than the Si 2p or Zr 3d peaks (around −0.2 eV). We speculate that this more pronounced shift could be attributed to the presence of thicker Na*_x_*PO*_y_* “islands” on the surface of NZSP_AS_ (Figure S6) leading locally to a larger surface potential. For the Na^0^|NZSP_polished_ interface (Figure 5c), the position of the peaks is seen to continuously shift in the first 100 min of plating before reaching more stable values.

The Si 2p and P 2p peak positions begin to shift after 150 min of plating (sometimes by more than 0.2 eV between two consecutive iterations). This scatter is a fitting artifact, which is caused by the difficulty in extracting a precise position for the peaks when their intensity is too low. The continuous shift of the peaks in the first 90 min of plating indicates that a surface potential is building up. This growing surface potential can be attributed to the growing thickness of the interphase. In other words, the evolution of the peak positions confirms that the interphase forms over a longer period for the Na^0^|NZSP_polished_ interface in comparison to the Na^0^|Na*_x_*PO*_y_*|NZSP interface. We invite the reader to refer to Part II of this study for more a more in-depth discussion on the origin of these peak shifts.^12^

### Aging Dynamics of Na^0^|NZSP Interfaces by Electrochemical Impedance Spectroscopy (EIS)

The operando XPS experiments established the protecting role of the thin Na*_x_*PO*_y_* layer on the surface of NZSP_AS_ samples against the formation of a thick SEI upon contact with Na^0^. The enhanced stability against Na^0^ of Na*_x_*PO*_y_*|NZSP surfaces in comparison to NZSP_polished_ ones is substantiated in this section by employing time-resolved EIS.

**Figure 6:**
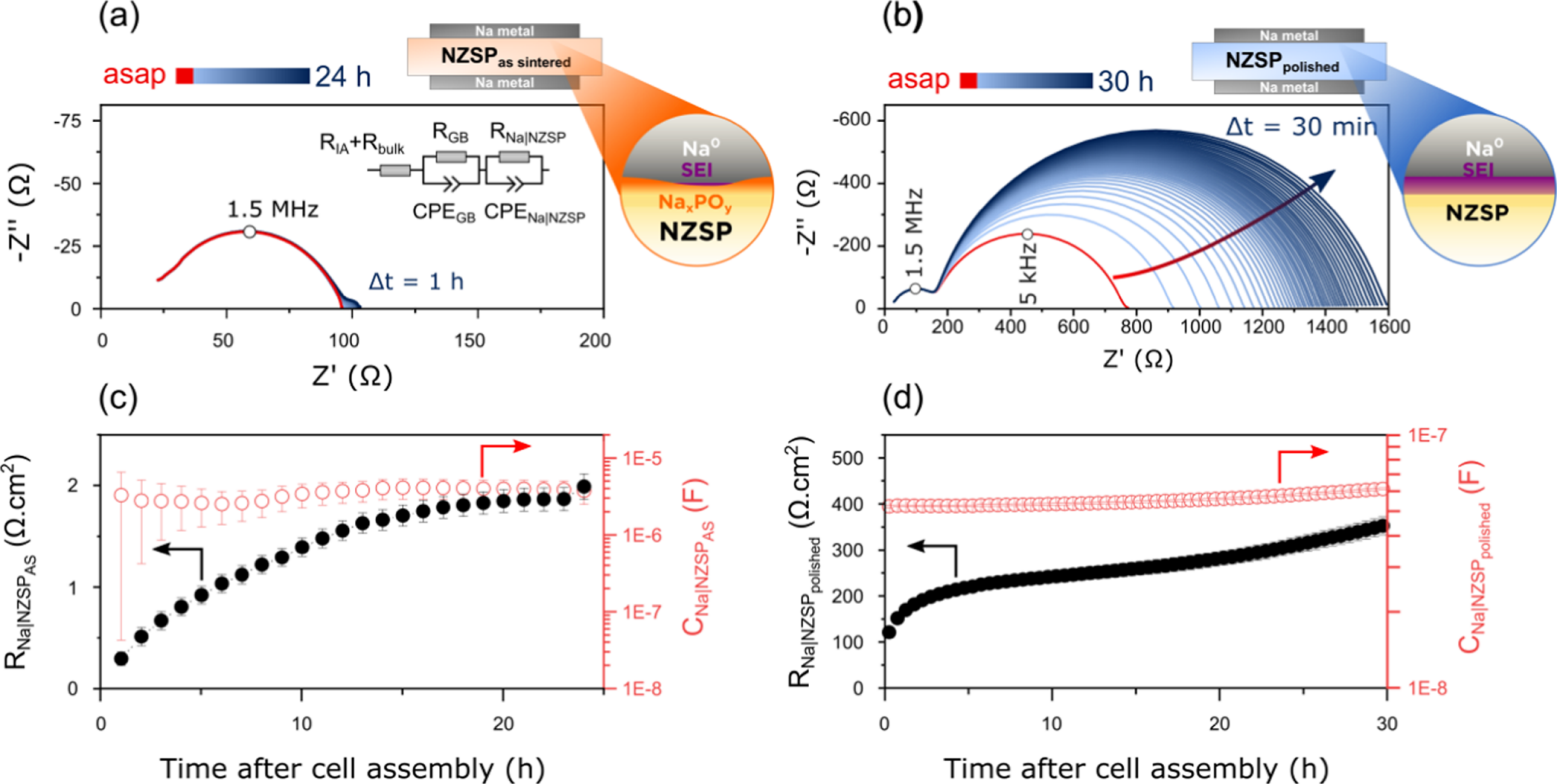
Time-resolved impedance spectra comparing the aging dynamics of two Na|NZSP|Na symmetrical cells assembled either with an as-sintered NZSP pellet (a) or a polished NZSP pellet (b). The equivalent circuit included in the inset of (a) was used to fit the impedance data (*R*_IA_ + *R*_bulk_: combined resistor accounting for the ohmic resistance of the impedance analyzer and the bulk resistance of the NZSP pellet; *R*_GB_ and CPE_GB_: grain boundary resistance and constant phase element; *R*_Na|NZSP_ and CPE_Na|NZSP_: Na|NZSP interface resistance and CPE). Evolution of the Na|NZSP_AS_ interface resistance and capacitance as a function of time (c). Evolution of the Na|NZSP_polished_ interface resistance and capacitance as *a* function of time (d). The cells were aged in a climate chamber at a temperature of 25 °C. Na metal electrode diameter: 8 mm in the cell with NZSP_AS_ and 7.2 mm in the cell with NZSP_polished_.

Two symmetrical cells consisting of an NZSP_AS_ or NZSP_polished_ pellet sandwiched between two Na^0^ electrodes were assembled and the evolution of their impedance was monitored over a little more than one day (with a time interval Δ*t* = 1 or 0.5 h respectively) at 25 °C. The impedance of the two cells is presented in Figure 6a,b in the form of Nyquist plots. The Nyquist plots were fitted to the equivalent circuit included in the inset in Figure 6a. As established in previous studies, the high-frequency semicircle (apex at 1.5 MHz) corresponds to grain boundary diffusion in the NZSP pellet.^14,27^ The second semicircle appearing at lower frequencies (apex at 5 kHz in Figure 6b) corresponds to the diffusion of Na^+^ ions across the Na^0^|NZSP interface. The initial interface resistance of the cell assembled with NZSP_AS_ is lower (0.3 ± 0.1 Ω·cm^2^) than the cell assembled with NZSP_polished_ (120 ± 7 Ω·cm^2^). This initial difference in interface resistance just after cell assembly was studied in more detail in a previous publication from our group.^14^

Regarding the evolution of the Na^0^|NZSP interface resistance as a function of time, Figure 6 shows that the increase is rapid in the first hours after cell assembly before stabilizing. This aging behavior suggests that the interphase between Na^0^ and NZSP is self-limiting, *i.e.*, that the decomposition species are electronically insulating (an interphase type commonly referred to as a SEI^4^). In addition, Figure 6 shows that the decomposition more strongly affects the cell assembled with NZSP_polished_ than the cell assembled with NZSP_AS_. In Figure 6c, the interface resistance of the NZSP_AS_ cell increased from 0.3 ± 0.1 to 2.0 ± 0.1 Ω·cm^2^ in 24 h, whereas in Figure 6d, the interface resistance of the NZSP_polished_ cell increased from 120 ± 7 to 360 ± 20 Ω·cm^2^ in 30 h.

The EIS results indicate that NZSP_AS_ samples are more stable against Na metal than NZSP_polished_ samples. We attribute this to the presence of a protective Na*_x_*PO*_y_* layer on the surface of NZSP_AS_ samples. Overall, the XPS and EIS results are aligned and demonstrate that a thicker and more resistive SEI forms at the Na^0^|NZSP_polished_ interface in comparison to the Na^0^|Na*_x_*PO*_y_*|NZSP one.

## Conclusions

Characterizing the decomposition reaction occurring at a buried interface with a technique whose depth resolution is limited to a few nanometers (such as XPS) is a challenging task. The operando experiment described in this study provides a lot of information in a single experiment and is therefore a very valuable tool in the characterization of alkali metal|SE interfaces. More precisely, the information which can be extracted from it includes the detection of new phases from the decomposition reaction at the interface; the equilibration time for the decomposition reaction; the rate of Na^0^ plating on the NZSP surface; and surface potentials associated with the growth of the interphase.

The first article of this two-part study focuses on establishing physically meaningful XPS fitting models for the Na^0^|NZSP interface while Na^0^ is being plated on the NZSP surface. For the Na 1s core level in particular, the models had to separate the signal emitted by the Na^0^ overlayer from the signals emitted by NZSP and the SEI. The study specifically focused on analyzing the kinetics of interphase growth and demonstrated that it can be monitored using widely available XPS instrumentation. The protocol established here is suitable to study the decomposition kinetics of a variety of systems evolving either to form stabilizing SEIs or continuously decomposing MIEC interphases. As the identification of the exact composition and structure of the Na^0^|NZSP SEI is limited with XPS due to overlapping contributions, we suggest that other characterization techniques such as cryogenic transmission electron microscopy (TEM) or atom probe tomography (APT) be employed in future studies.^28,29^

Beyond technique development, another important conclusion from the study is that as-sintered NZSP surfaces are more stable in contact with Na^0^ than polished NZSP surfaces. The presence of a Na*_x_*PO*_y_* layer on the surface of as-sintered NZSP pellets is acting as a self-formed protection layer against electrochemical decomposition. These results are also supported by time-resolved EIS.

In Part II of this study, data extracted from the XPS fittings are used to validate the applicability of the coupled ion electron transfer (CIET) theory to metal plating on solid electrolyte surfaces and to gain more detailed information about the kinetics of the plating reaction and SEI formation on Na*_x_*PO*_y_*|NZSP and NZSP_polished_ surfaces.

## Supporting Information Available

Evolution of the survey spectra of a Na metal sample as a function of sputtering time; residual gas contamination of Na metal; optical images of the surface of an NZSP pellet during operando plating taken with the in-built camera of the high-vacuum chamber of our XPS system; XPS fitting parameters of the sputter-cleaned Na metal; XPS fitting parameters of the Na^0^|Na_3_PO_4_|NZSP interface; and discussion about the flood gun settings and Na metal plating rate (PDF)

## Notes

The XPS and EIS raw data and fitting models used in this work are accessible on the following Zenodo repository: 10.5281/zenodo.6672033
