# Supplementary material for: Operando Characterization and Theoretical Modeling of Metal|Electrolyte Interphase Growth Kinetics in Solid-State Batteries. Part I: Experiments
Source: Chem Mater. 2023 Jan 20;35(3):853–62. doi: 10.1021/acs.chemmater.2c03130 (PMC9933420; doi:10.1021/acs.chemmater.2c03130)
Supplement: Supplementary file 1 — cm2c03130_si_001.pdf [file cm2c03130_si_001.pdf]

Supplementary information

Operando characterization and theoretical modelling of metal|electrolyte interphase growth kinetics in solid-state-batteries - Part I: experiments

Edouard Quérel,[1,*] Nicholas J. Williams,[1] Ieuan D. Seymour,[1] Stephen J. Skinner,[1] Ainara Aguadero,[1,2]

[1] Department of Materials, Imperial College London, Exhibition Road, London, SW7 2AZ, UK

[2] Instituto de Ciencia de Materiales de Madrid, ICMM-CSIC, Sor Juana Ines de La Cruz 3, 28049, Madrid, Spain

*Correspondence: edouard.querel@empa.ch

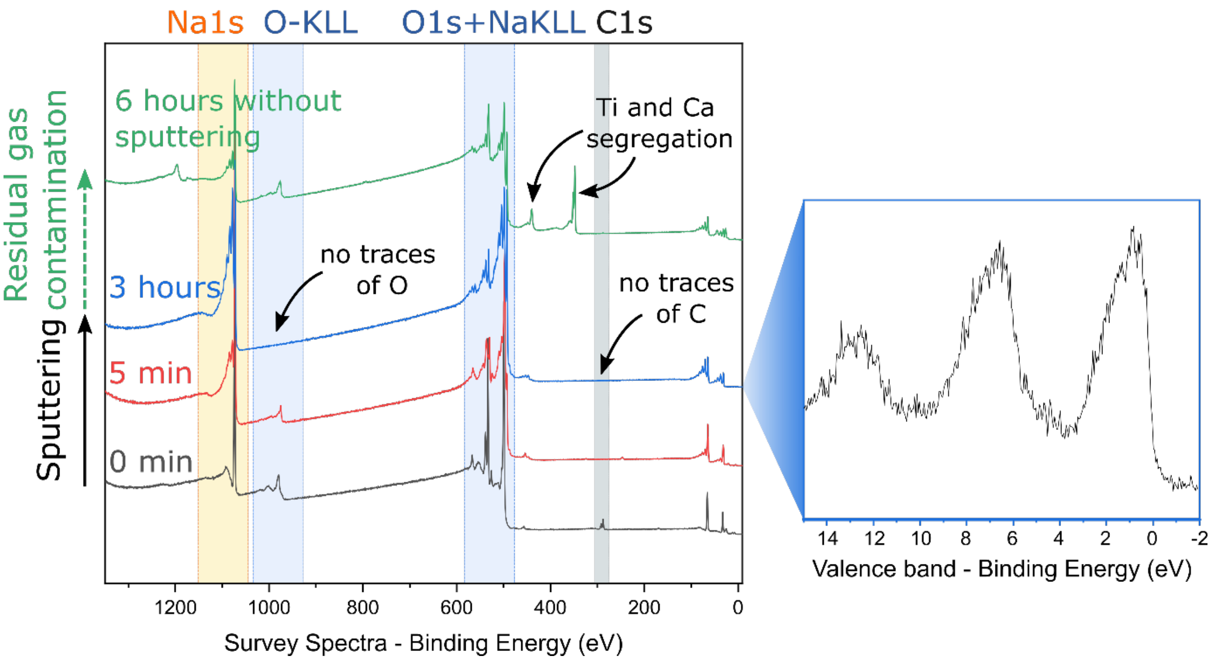

Figure S1 – Evolution of the survey spectra of a Na metal sample as a function of sputtering time. Despite having been prepared inside a clean glovebox and transferred to the XPS instrument in a vacuum transfer module, the surface of as-prepared Na metal foils (t = 0min) are passivated as indicated by the presence of C and O on the surface. Because Na-KLL photoelectrons are found in the same region as O1s photoelectrons, the O-KLL region is better to monitor the level of O contamination from the surface. Sputtering for 5 minutes is not enough to remove all traces of C and O from the surface. After 3 hours, all traces of contamination have disappeared. After that, the sample was left for 6 hours in the XPS chamber to assess the contamination from residual gases in the chamber. After 6 hours, a clear O signal is detected. Ca and Ti are also detected. The segregation of Ca (a contaminant commonly found in Na metal samples) to the surface during these 6 hours is a thermodynamically driven process with a reaction energy of -0.574 eV/atom for $Na_2O + Ca \rightarrow CaO + 2Na$ as extracted from the Materials Project.[1] Ti is present on the surface because the Ti sublimation pump of the instrument activated overnight during the pause. The slight misalignment of the valence band Fermi edge with the calibrated zero of the instrument was previously associated to the complexity of removing all contaminants on the non-sputtered side of the Na^0 foil (i.e. at the interface between the sample stage and Na^0 foil)[2]. Related to Figure 2.

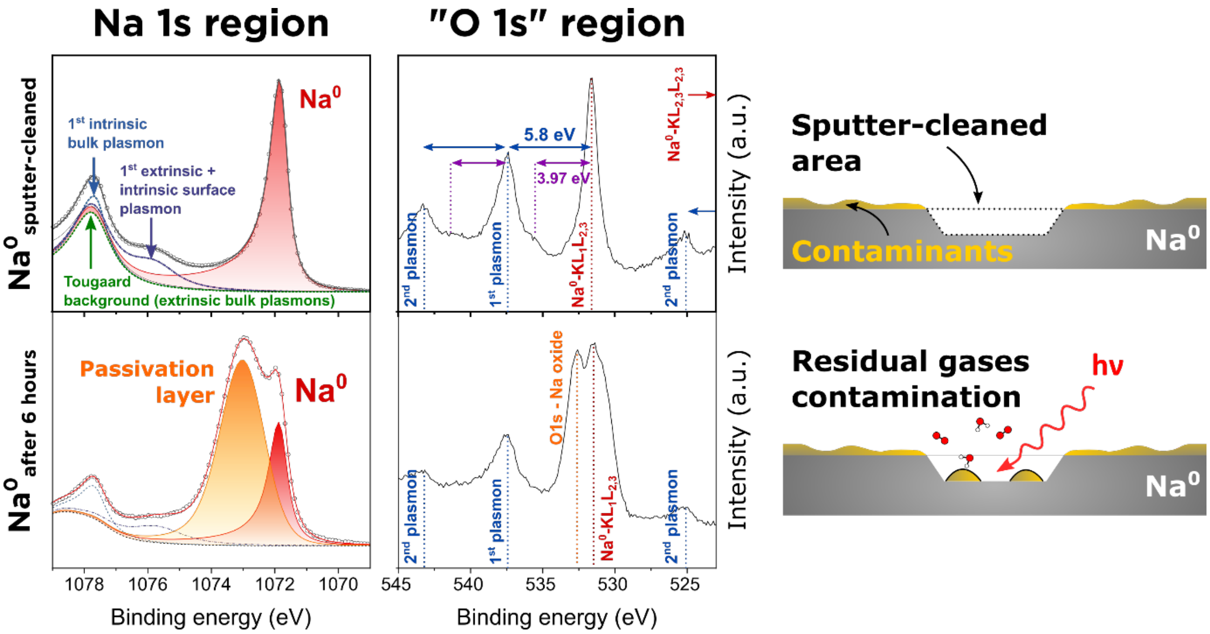

Figure S2 – Residual gas contamination of Na metal. Na1s and O1s XPS signals of a sputter-cleaned Na metal sample (top) and the same Na metal sample after 6 hours of exposure to the residual gases of the XPS chamber under ultra-high vacuum conditions. The Na1s photoelectron signal emitted by the species forming the passivation layer was modelled by a single peak with a wide FWHM. The formation of a passivation layer at the Na^0 surface is also indicated by a new peak at 532.6 eV in the O1s region. Establishing the exact composition of the passivating species which formed on the Na^0 surface was not required for this study. Related to Figure 2.

		Fitting			Constraints			
Region	Peak	BE (eV)	FWHM (eV)	Area	Line shape	BE (eV)	FWHM (eV)	Area
**Na1s**	Na0	1071.83	0.61	277172	LF(0.58,1.15,200,50)	1071.8 - 1071.9	<0.65	-
	1st extr.+intr. SP	1075.8	1.77	93174	//	1075.8-1075.85	-	-
	1st intr. BP	1077.61	0.59	23667	//	1077.61-1077.65	-	-
	Unidentified singlet	1079.8	2.92	100000	//	1079.8-1079.85	-	<100000
	1st extr. BP+SP	1081	3.93	18973	//	1081-1082	-	-

Table S1 – XPS fitting parameters of the sputter-cleaned Na metal sample. extr./intr. = extrinsic/intrinsic. SP = surface plasmon, BP = bulk plasmon. Related to Figure 2.

		Fitting			Constraints			
Region	Peak	BE (eV)	FWHM (eV)	Area	Line shape	BE (eV)	FWHM (eV)	Area
Na1s	NZSP	1073.94	1.59	227806	LA(1.25,1.53,243)	-	-	-
P2p	3/2 NZSP	135.66	1.36	4448	LA(1.53,243)	-	-	-
	1/2 NZSP	136.53	1.36	2224	//	P2p3/2 + 0.87	P2p3/2 *1	P2p3/2*0.5
Si2p	3/2 NZSP	104.2	1.56	3500	LA(1.53,243)	-	-	-
	1/2 NZSP	104.8	1.56	1750	//	Si2p3/2 +0.6	Si2p3/2*1	Si2p3/2*0.5
Zr3d	5/2 NZSP	185.09	1.37	22059	LA(1.53,243)	-	-	-
	3/2 NZSP	187.49	1.37	14706	//	Zr3d5/2 (NZSP) + 2.4	Zr3d5/2 (NZSP)*1	Zr3d5/2 (NZSP)*0.667

Table S2 – XPS fitting parameters of the reference $NZSP_{polished}$ sample. Related to Figure 3.

		Fitting			Constraints			
Region	Peak	BE (eV)	FWHM (eV)	Area	Line shape	BE (eV)	FWHM (eV)	Area
Na1s	Na0	1071.56	0.66	160516	LF(0.58,1.17,200,85)	-	0.6 - 0.75	-
	Surf. plasmon	1075.38	1.84	97758	//	1075.2 - 1075.6	1.6 - 2	-
	Bulk plasmon	1077.37	1.13	75114	//	1077 - 1079	0.8 - 1.2	-
	NZSP	1073.47	1.45	55030	LA(1.53,243)	1073.2 - 1073.8	1.45 - 1.65	Fixed - same Na:Si ratio as ref. sample
	int. & pass.	1073.05	1.97	136388	LA(1.53,243)	-	-	-
P2p	3/2 NZSP	135.09	1.27	1018	LA(1.53,243)	-	-	-
	1/2 NZSP	135.96	1.27	509	LA(1.53,243)	P2p3/2 + 0.87	P2p3/2 *1	P2p3/2*0.5
Si2p	3/2 NZSP	103.55	1.51	858	LA(1.53,243)	-	-	-
	1/2 NZSP	104.15	1.51	429	LA(1.53,243)	Si2p3/2 +0.6	Si2p3/2*1	Si2p3/2*0.5
Zr3d	5/2 NZSP	184.55	1.50	3246	LA(1.53,243)	184 -184.55	1 - 1.5	-
	3/2 NZSP	186.95	1.50	2164	LA(1.53,243)	Zr3d5/2 (NZSP) + 2.4	Zr3d5/2 (NZSP)*1	Zr3d5/2 (NZSP)*0.667
	5/2 int. 1	183.66	1.50	2159	LA(1.53,243)	183 -183.7	Zr3d5/2 (NZSP)*1	-
	3/2 int. 1	186.06	1.50	1439.02	LA(1.53,243)	Zr3d5/2 (SEI1) + 2.4	Zr3d5/2 (NZSP)*1	Zr3d5/2 (SEI1)*0.667
	5/2 int. 2	182.24	1.50	434.607	LA(1.53,243)	181 - 183	Zr3d5/2 (NZSP)*1	-
	3/2 int. 2	184.64	1.50	289.738	LA(1.53,243)	Zr3d5/2 (SEI2) + 2.4	Zr3d5/2 (NZSP)*1	Zr3d5/2 (SEI2)*0.667
Background:<br>- the Na1s region was fitted with a Tougaard background (U 4 Tougaard: 0.8, 33, 0.8, 0). The first cross-section term (which modulates the intensity of the background) was optimized for each plating cycle to minimize residuals<br>- all other peaks were fitted with a Shirley type background								

Table S3 – XPS fitting parameters of the Na^0|$NZSP_{polished}$ interface (dataset collected after 91 minutes of plating). Related to Figure 3.

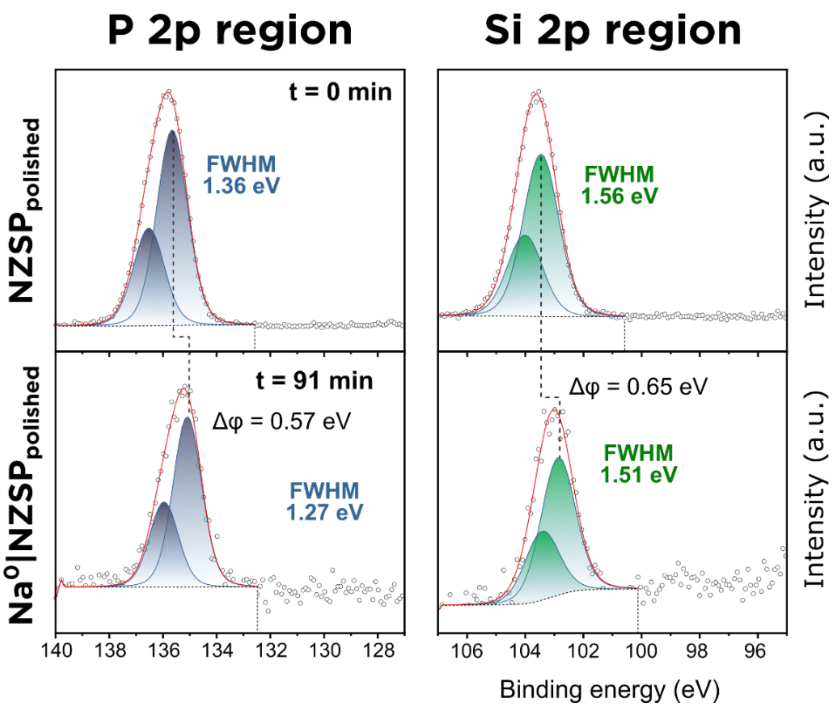

Figure S3 – The P2p and Si2p core level regions of the $Na^0|NZSP_{polished}$ interface compared with those of the reference $NZSP_{polished}$ sample.

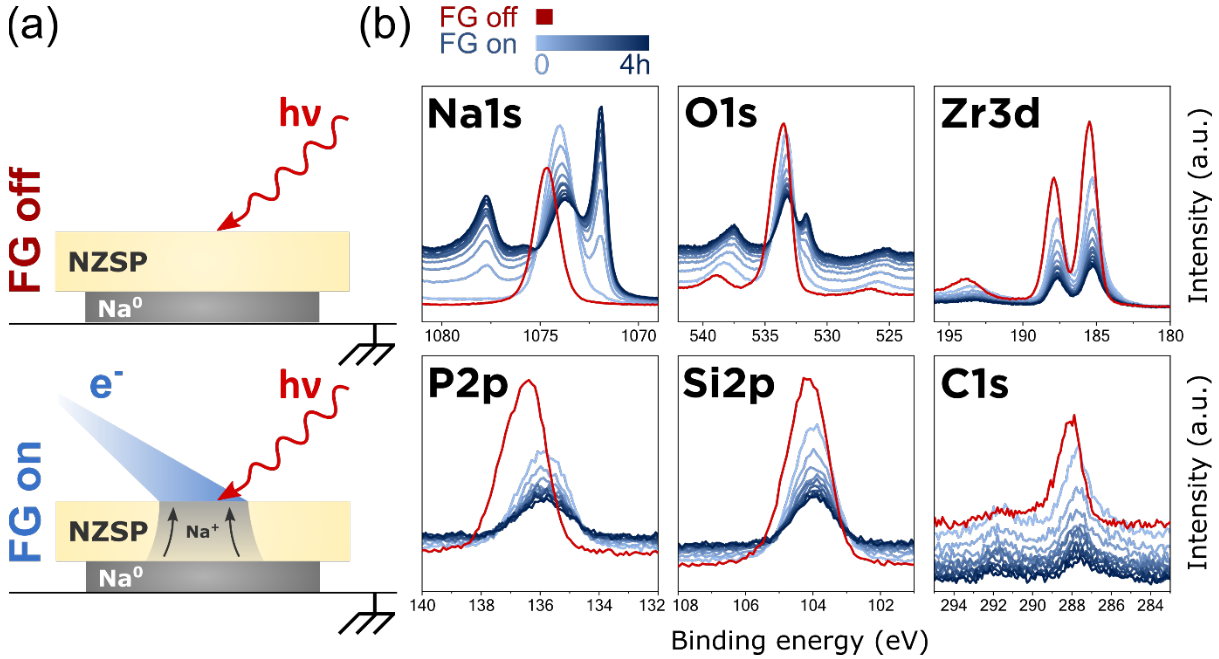

Figure S4 – ***Operando*** **plating of Na^0 on a $NZSP_{AS}$ surface inside the XPS.** (a) Schematic representation of the two XPS analysis configurations; (b) Evolution of selected core level regions with increasing Na^0 plating time. An initial set of data (in red) was measured with the FG off (reference signal from $NZSP_{AS}$). The following spectra (in shades of blue) were measured in an iterative loop (each iteration lasted 33 minutes).

		Fitting			Constraints			
Region	Peak	BE (eV)	FWHM (eV)	Area	Line shape	BE (eV)	FWHM (eV)	Area
Na1s	NZSP	1074.63	1.4	166765	LA(1.15,1.4,243)	-	-	-
P2p	3/2 NZSP	136.29	1.31	3830	LA(1.53,243)	-	-	-
	1/2 NZSP	137.16	1.31	1915	//	P2p3/2 + 0.87	P2p3/2 *1	P2p3/2*0.5
Si2p	3/2 NZSP	103.98	1.19	3659	LA(1.53,243)	-	-	-
	1/2 NZSP	104.58	1.19	1829	//	Si2p3/2 +0.6	Si2p3/2*1	Si2p3/2*0.5
Zr3d	5/2 NZSP	185.5	1.24	25307	LA(1.53,243)	-	-	-
	3/2 NZSP	187.9	1.24	16872	//	Zr3d5/2 (NZSP) + 2.4	Zr3d5/2 (NZSP)*1	Zr3d5/2 (NZSP)*0.667

Table S4 – XPS fitting parameters of the reference $NZSP_{AS}$ sample. Related to Figure 4.

		Fitting			Constraints			
Region	Peak	BE (eV)	FWHM (eV)	Area	Line shape	BE (eV)	FWHM (eV)	Area
Na1s	Na0	1071.89	0.64	63006	LF(0.61,1.17,200,70)	-	0.64	-
	Surf. plasmon	1075.84	2.00	43054	//	-	-	-
	bulk plasmon	1077.73	1.20	35139	//	1077-1080	-	-
	NZSP	1073.87	1.71	62109	LA(1.53,243)	-	-	Fixed - same Na:Si ratio as ref. sample
	int. & pass.	1074.10	1.71	10507	//	1073-1074.5	-	-
P2p	3/2 NZSP	135.64	1.62	1620	LA(1.53,243)	-	-	-
	1/2 NZSP	136.51	1.62	810.1	//	P2p3/2 + 0.87	P2p3/2 *1	P2p3/2*0.5
Si2p	3/2 NZSP	103.80	1.23	1793	LA(1.53,243)	-	-	-
	1/2 NZSP	104.40	1.23	896	//	Si2p3/2 +0.6	Si2p3/2*1	Si2p3/2*0.5
Zr3d	5/2 NZSP	185.28	1.2	9747	LA(1.53,243)	-	-	-
	3/2 NZSP	187.68	1.20	6498	//	Zr3d5/2 (NZSP) + 2.4	Zr3d5/2 (NZSP)*1	Zr3d5/2 (NZSP)*0.667
	5/2 int. 1	184.11	1.20	1366	//	-	Zr3d5/2 (NZSP)*1	-
	3/2 int. 1	186.51	1.20	911	//	Zr3d5/2 (SEI1) + 2.4	Zr3d5/2 (NZSP)*1	Zr3d5/2 (SEI1)*0.667
	5/2 int. 2	183.00	1.20	377	//	-	Zr3d5/2 (NZSP)*1	-
	3/2 int. 2	185.40	1.21	251	//	Zr3d5/2 (SEI2) + 2.4	Zr3d5/2 (NZSP)*1	Zr3d5/2 (SEI2)*0.667
Background:<br>- the Na1s region was fitted with a Tougaard background (U 4 Tougaard: 1.45, 33, 0.8, 0). The first cross-section term (which modulates the intensity of the background) was optimized for each plating cycle to minimize residuals<br>- all other peaks were fitted with a Shirley type background								

Table S5 – XPS fitting parameters of the Na^0|Na_3PO_4|NZSP interface (dataset collected after 96 minutes of plating). Related to Figure 4.

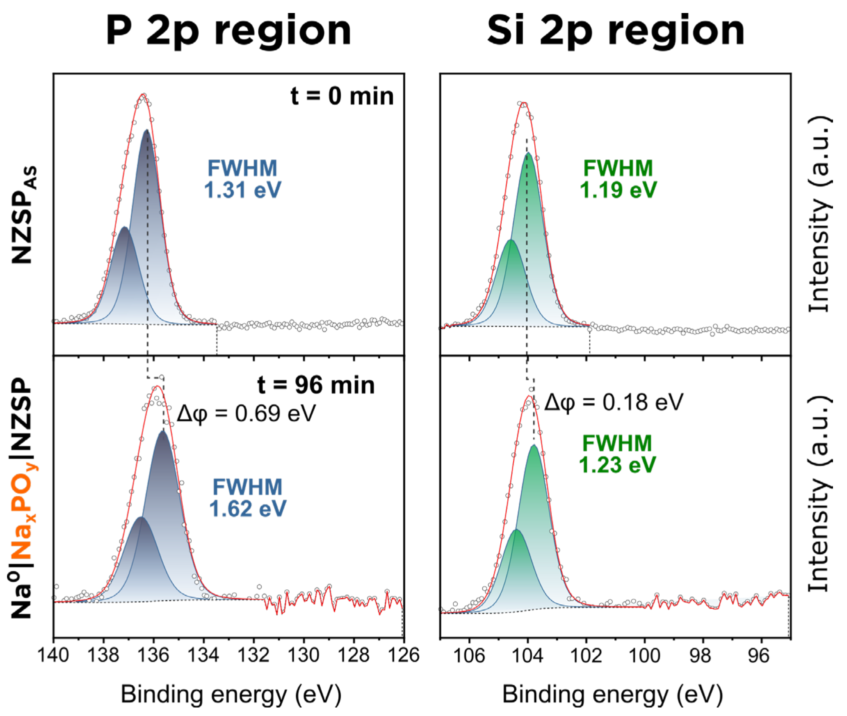

Figure S5 – The P2p and Si2p core level regions of the Na^0|Na_3PO_4|NZSP interface compared with those of the reference $NZSP_{AS}$ sample.

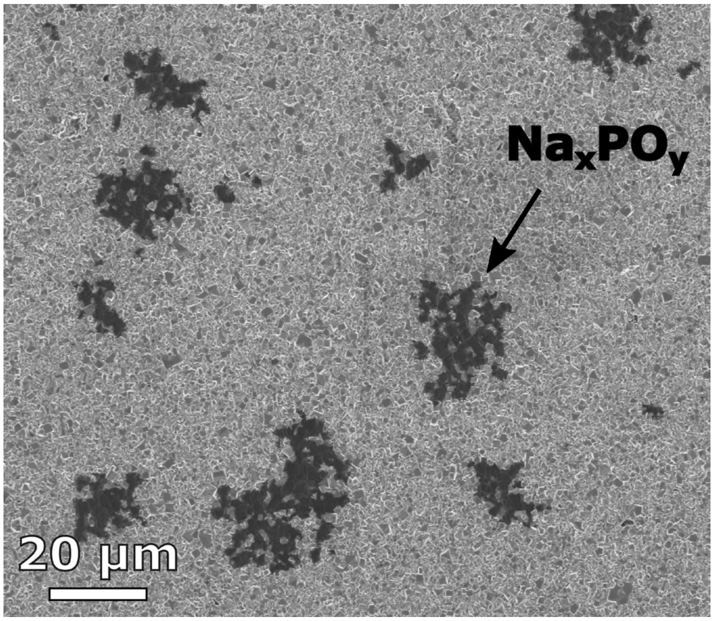

Figure S6 – SEM micrograph of a $NZSP_{AS}$ surface collected in secondary electron mode using the in-lens detector of a Zeiss Leo Gemini 1525 at a working distance of 7 mm with an electron beam accelerating voltage of 5 kV and a 30 µm aperture. The composition of the darker islands was identified to be a sodium phosphate phase by EDX in a previous study.[3] Related to Figure 6.

Electron flood gun settings and Na metal plating rate

The area covered by the flood gun was extrapolated from the area covered by Na metal at the end of the experiment in Figure S7 (assuming the area is a circle of roughly 6 mm in diameter). Thus, with a ~5 μA current, the current density should be around 17.8 μA cm^{-2}. Considering the capacity of Na metal which is 1131 mAh cm^{-3}, this should result in a plating at a rate of 158 nm h^{-1} (2.63 nm min^{-1}) if all the electrons participate to the plating reaction.

From these calculations and knowing that the escape depth for photoelectrons is typically around 5 nm for an XPS instrument with an Al Kα source, there is legitimate reasons to emit some concerns about why we can still observe an electrolyte signal after 4 h of plating (for instance in Figure S4). We believe that several reasons can explain this paradox: (1) not all flood gun electrons participate to the plating reaction. There is a competition for electrons between the plating reaction and the refilling of the holes produced by the photoemission process. We believe that the majority of flood gun electrons serve their original purpose of replenishing holes. Thus, the actual plating current is much lower than the instrument set current, This competition for electrons makes it hard to know the actual plating current from the instrument set current. (2) It was quite difficult to center the flood gun concentrically with the XPS analysis area with our instrument. Thus, the flood gun was often slightly offset (as can be seen in Figure S7 below) and therefore the current density was maybe slightly different than in the middle of the flood gun area.

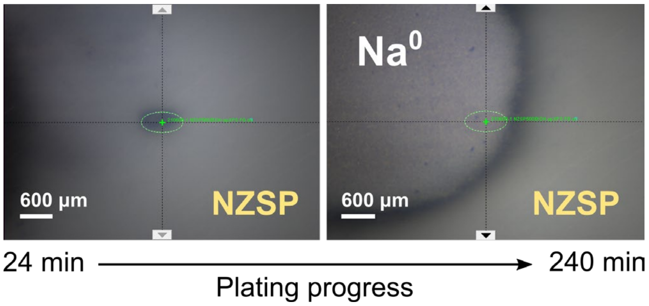

Figure S7 – Optical images of the surface of a NZSP pellet during *operando* plating taken with the in-built camera of the high-vacuum chamber of our XPS system. The image clearly shows the area where Na metal plated and that the X-ray spot area is slightly off the centre of this area.

References

1. Jain, A. *et al.* Commentary: The materials project: A materials genome approach to accelerating materials innovation. *APL Materials* 1, 011002 (2013).
2. Wood, K. N. & Teeter, G. XPS on Li-Battery-Related Compounds: Analysis of Inorganic SEI Phases and a Methodology for Charge Correction. *ACS Appl. Energy Mater.* 1, 4493–4504 (2018).
3. Querel, E. *et al.* The role of NaSICON surface chemistry in stabilizing fast-charging Na metal solid-state batteries. *J. Phys. Energy* 3, (2021).
